# A network-based approach to model volcanic repose durations

**DOI:** 10.1038/s41598-026-56253-7

**Published:** 2026-06-10

**Authors:** Elia Altimani, Catherine Annen, Rémy Cazabet

**Affiliations:** 1 Institute of Geophysics of the Czech Academy of Sciences, Prague, Czech Republic; 2 Université Claude Bernard Lyon 1, CNRS, INSA Lyon, LIRIS, UMR5205, Villeurbanne, F-69622 France

**Keywords:** Natural hazards, Solid Earth sciences

## Abstract

The global distributions of volcanic eruption durations and of repose times between two eruptions have broad and heterogeneous shapes. Statistical analyses indicate that both distributions may exhibit a power-law tail for medium-to-long timescales. This scale-free behaviour could be symptomatic of volcanic systems that self-organize into a critical state. In this paper, we build on a former model for magma ascent through a pipe of stacked cells, which successfully reproduces the trend of the global distribution of volcanic eruption durations, but does not account for the inter-event times between eruptive episodes. By adapting and implementing this model within tree-like graphs instead of a linear structure, we are able to retain the behaviour of the eruption duration distribution, while obtaining a broad distribution for the repose times. In contrast with the pipe linear structure, the tree-like structure accounts for the fact that a large part of the magma that ascends through the crust does not reach the surface. This result highlights the importance of modelling volcanic plumbing systems as networks that reflect their complexity and variety.

**Supplementary Information:**

The online version contains supplementary material available at 10.1038/s41598-026-56253-7.

## Introduction

Volcanic plumbing systems are complex systems^1^. They consist of networks of magma reservoirs, dikes, and conduits, whose dynamics are governed by non-linear processes that occur across a wide range of spatial and temporal scales. Eruptions and their variability, for instance in terms of duration, are just the culminating expression of this complexity and can last from a few hours to hundreds of years^2^.

In the literature, several studies have investigated the distribution of eruption durations, focusing either on specific eruption types (e.g., lava dome eruptions^3,4^) or on individual volcanic systems, (e.g., Etna^5,6^ and Piton de la Fournaise^7)^. These studies often report distributions compatible with scale-free behaviour; however, results obtained at the scale of individual volcanoes are not necessarily consistent with those derived from global datasets, reflecting the heterogeneity of volcanic systems. At the global scale, eruption duration distributions have also been interpreted as exhibiting power-law tails^6^, although alternative statistical models may provide comparable fits, and no clear consensus has yet been reached.

The statistical behaviour of repose times, i.e., the inter-event times between two eruptions, remains much more debated. Godano & Civetta (1996) showed that the eruptive history of Vesuvius between the 17th and 20th centuries is globally consistent with volcanic eruptions being the results of a Poissonian process^8^, which is memoryless, i.e., the probability of an eruption to happen does not depend on the time elapsed since the previous one. This would lead to a distribution of repose durations with an exponential decay. In contrast, Grasso & Bachèlery (1995) showed that the repose durations of Piton de la Fournaise during the 20th century follow a broken power-law distribution^7^. More recently, Sanchez et al. (2012) analyzed the repose-duration distributions of the most active volcanoes on Earth and identified a unified scaling law consistent with a log-normal distribution^9^, in contrast to the classification proposed by Marzocchi et al. (2003), who distinguished between closed- and open-conduit volcanoes based on the statistical behavior of repose durations^10^.

Power laws and, more generally, heavy-tailed distributions characterize phenomena emerging from complex systems that self-organize near a critical state. For this reason, Cannavò & Nunnari (2016) developed a toy model for magma ascent through a pipe, conceptually inspired by the sand-pile model^11^. Similar threshold-based models have been widely used in seismology, such as the block–spring model originally introduced to reproduce the statistical properties of earthquake occurrence^12^.

In the model by Cannavò & Nunnari (2016), at each time step, magma is input from below in the form of discrete batches. Its propagation through a series of vertically stacked cells is governed by a stochastic process with a threshold mechanism. The stochastic component of the dynamics allows for symmetric upward and downward movements, which could mimic local convective-like motions of magma, related to density gradients due to temperature fluctuations or compositional fractionation^13,14^. The threshold mechanism instead introduces a bias for upwards movement, so that magma travels on average upwards. Magma exiting at the top of the pipe is constrained to one batch per time step and is considered erupted, so that eruptive episodes and repose periods can be identified as continuous intervals during which the output remains unchanged. This constraint effectively defines eruption duration in terms of the number of erupted magma batches, implicitly assuming a constant mass discharge rate during eruptive phases. This model successfully reproduces the scale-free behaviour of the distribution of eruption durations, but does not account for the repose times.

In the first part of the paper, we analyse both eruption durations and repose durations using the Smithsonian Institution’s Global Volcanism Program (GVP) dataset^2^ updated up to 2020. Due to the limited size and incompleteness of the database, we do not claim that the observed distributions strictly follow a specific statistical law. Rather, our analysis aims to assess qualitative consistency with previous studies on eruptions^6^, characterize the global behaviour of repose periods and, finally, allow us to compare our model with observed data.

In the second part of the paper, we generalize the Cannavò & Nunnari (2016) model by replacing the linear structure with tree-like graphs obtained through a rewiring procedure. Graphs are useful mathematical tools, commonly used to represent both artificial and natural systems^15^, as they capture the structural complexity of interactions among the elements of a system. The aim of this extension is to introduce variability in the structure of volcanic plumbing systems, which may be relevant to reproduce the diversity of volcanic behaviour observed at a global scale. We acknowledge that the original model also involves simplifying assumptions, such as the one-erupted-batch-per-timestep rule and the possibility of downward movements, which may seem artificial. However, in order to isolate the effect of structural complexity on the system’s behaviour, we focus here exclusively on modifying the network topology, leaving the dynamical rules unchanged, apart from the needed adaptations. The extension allows magma to propagate along branching pathways that account for the possibility of temporary storage in different regions of the crust, making the model more realistic. For certain values of its parameters, the model preserves the power-law behaviour of eruption durations, while producing broad repose-period distributions spanning several orders of magnitude, in closer agreement with observations.

## Results

### Data analysis

In this section, we summarize the main statistical patterns in eruption and repose durations, compare them qualitatively with previous studies, and extract baseline estimates used later to evaluate our model.

We analysed the global record of volcanic eruptions compiled by the Smithsonian Institution’s Global Volcanism Program^2^. The dataset contains 4005 confirmed eruptions from 451 volcanoes worldwide for which at least the start and end years are known. From this dataset, we extracted the duration of each eruptive episode and the repose intervals between consecutive eruptions of the same volcano (see Methods for details on data processing and uncertainty treatment). The dataset is incomplete: historical records become increasingly sparse the further back in time we look, leading to gaps in information on volcanic activity. However, our statistical analysis is supported by Monte Carlo simulations that sample subsets of the dataset in order to assess the robustness of the results in the presence of missing data.

**Fig. 1:**
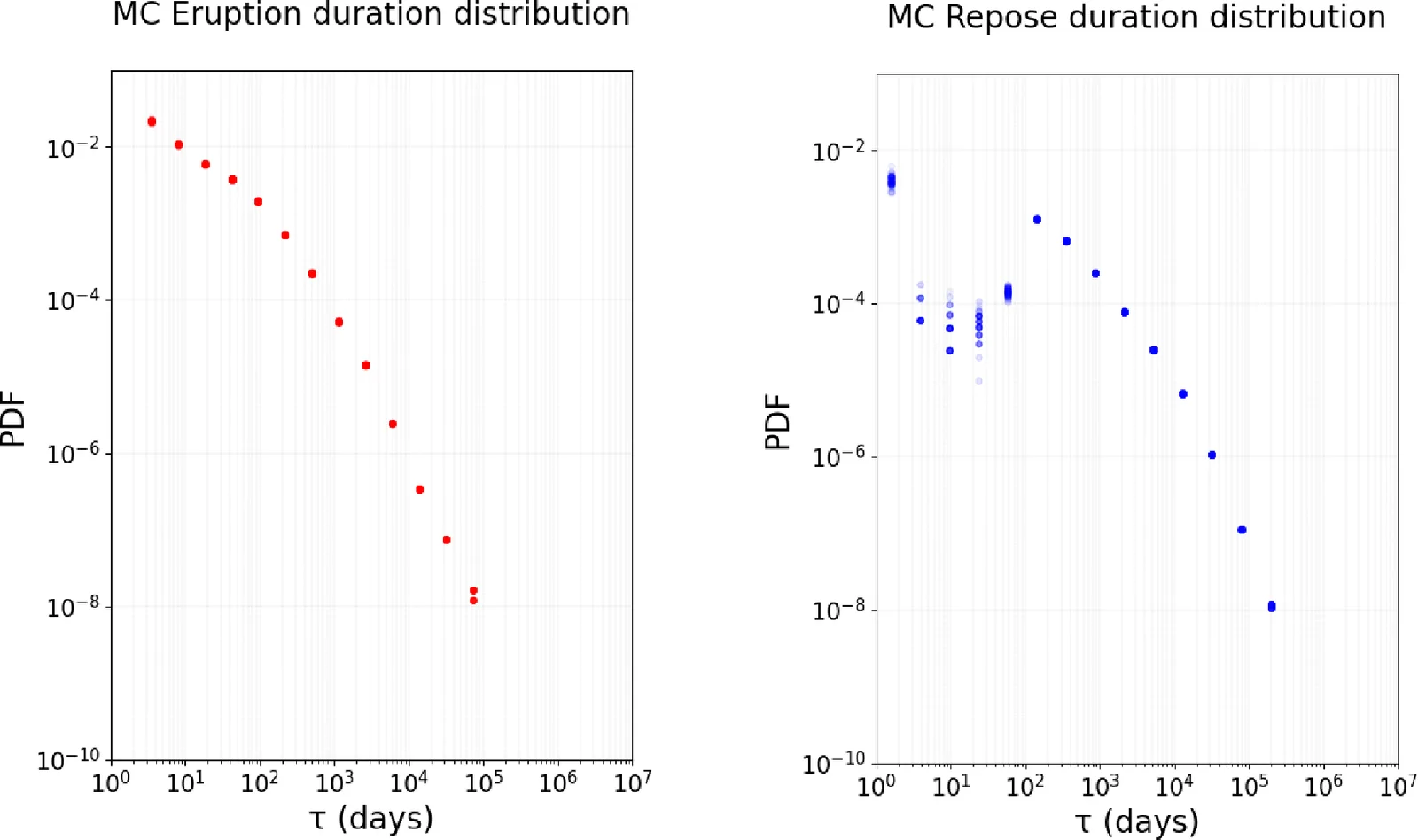
Probability Density Functions (PDFs) of eruption (left) and repose (right) durations, reconstructed through Monte Carlo (MC) simulations to account for the uncertainties associated with the data. The anomalies in the shapes of both distributions for short durations ($\:<1{0}^{2}$) are probably caused by undersampling of repose durations and consequent overestimation of eruption durations, due to the definition of ongoing eruptions used in the creation of the dataset, as explained in the text.

In Fig. 1, we show the Probability Density Functions (PDFs) reconstructed from the dataset, by running Monte Carlo simulations to account for the uncertainties related to each duration (see Methods section). The distributions are marked by high heterogeneity, displaying a heavy tail for durations above $\:{\:\sim10}^{3}$ days. The different shapes of both distributions up to that point may reflect the difficulty in defining the start and the end of an eruption. When only a few days or months separate two eruptive episodes, it is unclear whether they should be considered distinct events or phases of the same eruption. In the database, eruptive episodes separated by less than 3 months are most of the time considered as the same ongoing eruption, unless there’s clear evidence that allows a distinction between the two events^16^. This ambiguity leads to an undersampling of short repose periods and an overestimation of eruption durations. There is also an upper limit inherent in the limitedness of the dataset we considered, in which the oldest eruption dates back to 10 CE. This imposes a cut-off at $\:{\sim7\times\:10}^{5}$ days, so that the rarest and longest events may be underrepresented. Furthermore, recording completeness decreases progressively backward in time, making older eruptions increasingly unlikely to be documented^17^.

We looked for the best fit for both distributions. With respect to the procedure by Cannavò & Nunnari (2016), we directly fit raw data, since our aim is not to obtain a precise statistical analysis but to determine the behaviour of these distributions. We ran 1000 Monte Carlo simulations, in which, each time, we retained only a subset of the data. We also replicated the experiment for eruption durations, so that we can test the robustness of our approach by comparing our results on eruption durations to those of Cannavò & Nunnari (2016) and then apply it to repose times.

**Fig. 2:**
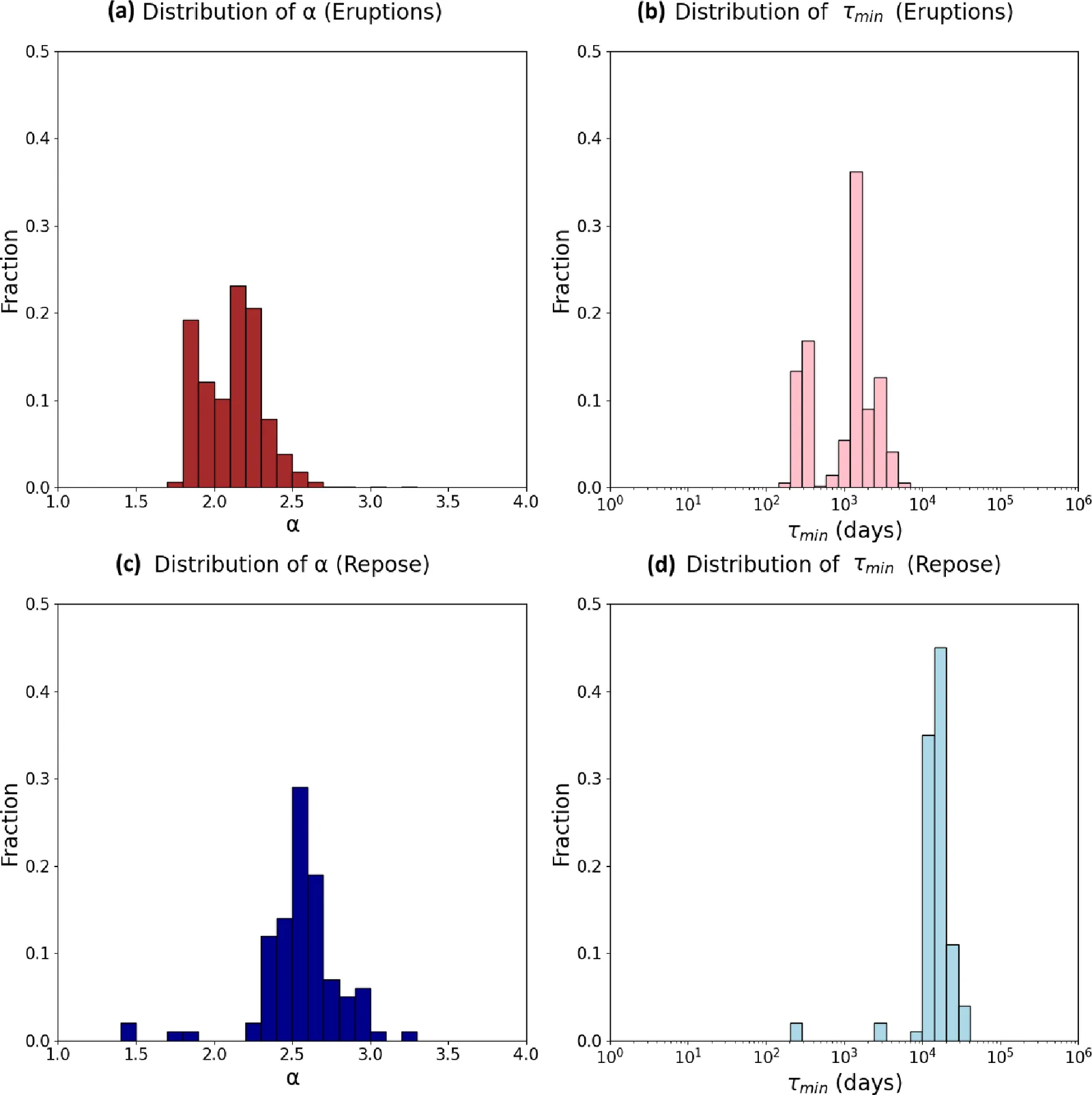
Distributions of exponents **(a**,** c)** and optimal minima **(b**,** d)** of the power-law fits obtained through Monte Carlo simulations, in which only a portion of the dataset is retained in each run. Top: eruptions; bottom: repose periods.

**Fig. 3:**
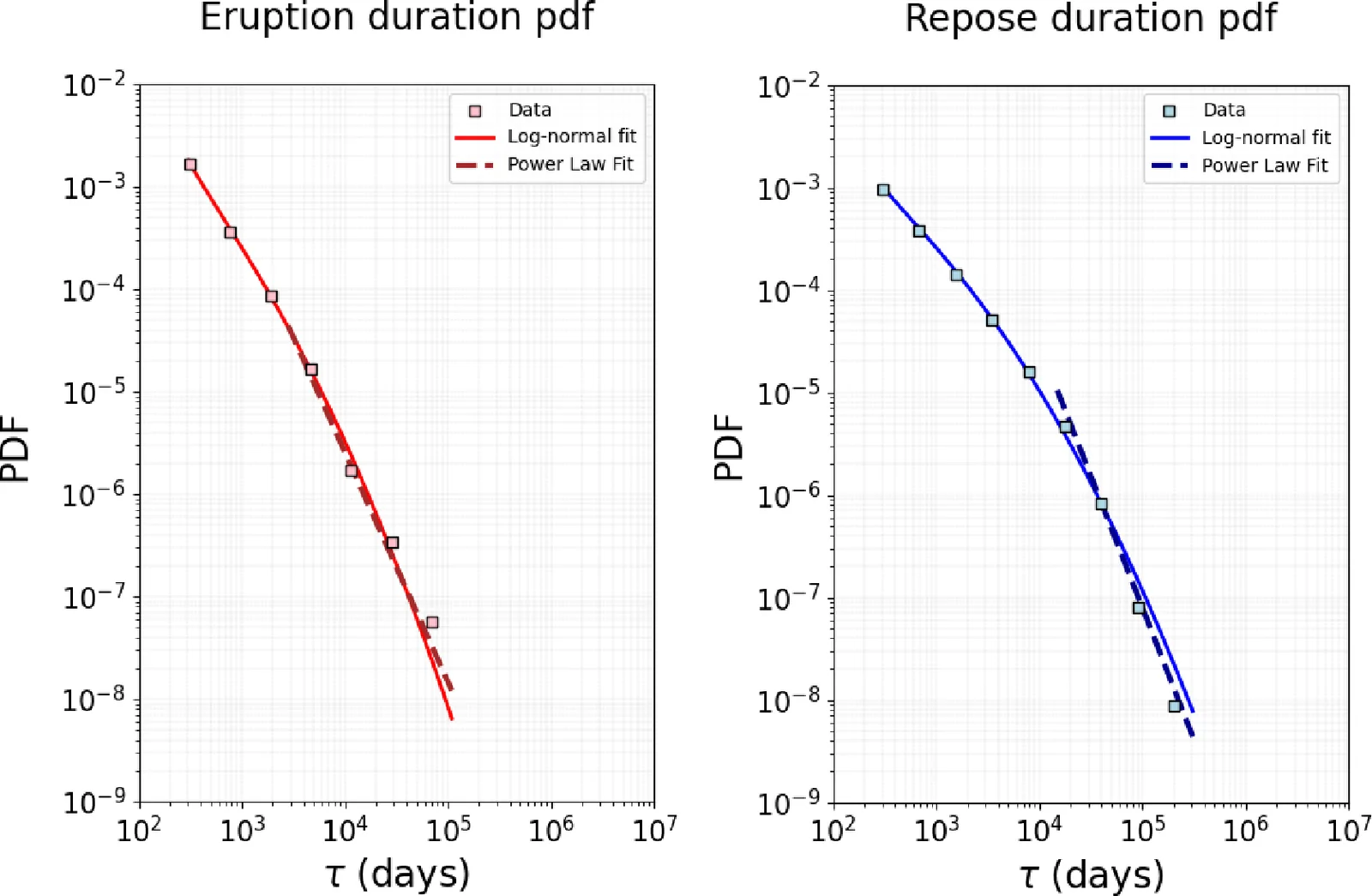
Best power-law and log-normal fits for eruption durations (left) and repose times (right) compared to the data PDFs. The temporal axis is limited to the range of durations not affected by the possible undersampling of repose times due to the 3-months rule inherent in the dataset and explained in the text.

For eruption durations, the average exponent of the power-law fit is $\:{{\alpha\:}^{e}}_{new}=2.12\:\pm\:\:0.19$ (Fig. 2a) and the average minimum duration that optimizes the fit is$\:\:{\tau\:}_{min\:,new}^{e}=1485\:\pm\:\:1043$ days (Fig. 2b). Both values are close to the ones found by Cannavò and Nunnari (2016), $\:{{\alpha\:}^{e}}_{CN}=2.16\:\pm\:\:0.22$ and $\:{\tau\:}_{min,\:CN}^{e}=1782\:\pm\:\:581$ days. For repose periods, the average exponent of the power-law fit is $\:{\alpha\:}^{r}=2.56\:\pm\:\:0.27$ (Fig. 2c), and the average minimum duration that optimizes the fit is $\:\:{\tau\:}_{min}^{r}=15903\:\pm\:\:6186$ days (Fig. 2d). Performing a Kolmogorov-Smirnov (KS) test, all fits give * p*-value > 0.1, so that the power-law hypothesis cannot be rejected. Here, * e* denotes eruptions, * r* denotes repose, * new* refers to results obtained in this study and * CN* to those reported by Cannavò and Nunnari (2016). We show the fitted curves in Fig. 3, where also alternative fits with log-normal distributions are displayed (see Discussion and Methods sections).

In the next section we build on the pipe model developed by Cannavò and Nunnari (2016) and modify it in order to produce also a broad distribution for repose durations. For the final quantitative comparison between model predictions and empirical data, we consider the power-law exponent $\:\alpha\:$, which, because of the scale-free nature of the power-law distribution, allows comparison with an abstract model independent of physical timescales.

### Pipe model

Cannavò & Nunnari (2016)^6^ developed a model (hereon, called the pipe model) inspired by the sand-pile mode^11^. The sand-pile model is a paradigmatic example of a complex system that exhibits Self-Organizing Criticality (SOC) and produces scale-free, power-law distributions of event sizes, which may be the underlying nature of volcanic plumbing systems^18^. This model has been adopted and adapted to describe many natural phenomena exhibiting power-law statistics: from solar flares^19,20^ and earthquakes^12^ to neuronal avalanches^21^ and the spatial organization of rainforests^22^.

Here, we review the fundamental mechanisms underlying the pipe model, reframing its structural component in the context of graph theory. A graph is a mathematical object made up of entities (nodes) that may have connections (edges) with one another. In this case, nodes would represent different regions of the crust where magma can be stored and edges would be dikes and conduits through which magma travels. Cellular automaton models for volcanic and magmatic systems have already been used in the past^23,24^, but the proposal of graph structures is a quite recent development^1,25^.

The structure of the original model built by Cannavò & Nunnari (2016) is a vertical pipe of stacked cells that can be represented as a chain, i.e., an undirected graph * G=(V,E)* of * n+1* nodes, where $\:V=\{0,\:1,\dots\:,n\}$ and $\:\:E=\left\{\left(i,i+1\right)\:\right|\:i\in\:\{0,\:1,\:\dots\:,\:n-1\}\}$. Apart from the source (bottom node, $\:\:0$) and the volcano (top node, $\:\:n$), each node * i* has two neighbours: one above, $\:\:i+1$, and one below, * i-1*.

At each time step $\:\:t$, the source node receives a constant input $\:{I}_{0}$ of magma and each node * i* is characterized by a quantity of magma, $\:\:{Q}_{i}\left(t\right).$ The magma’s dynamics are governed by a stochastic process with a threshold mechanism, related to a critical value of the volume of magma, $\:\:{Q}_{c},$ that in the original model is taken equal to * 1*. When $\:\:{Q}_{i}\left(t\right)<\:{Q}_{c}$, a random number $\:{\rho\:}_{i}\left(t\right)\in\:[-\mathrm{1,1}]$ is generated. Its absolute value represents the fraction of mobile magma that is being transported away from node * i* at time * t* and its sign gives the direction of movement: upwards or downwards for positive and negative sign, respectively.

When $\:\:{Q}_{i}\left(t\right)>\:{Q}_{c}$, the same stochastic process holds, but only after having moved a quantity $\:{Q}_{c}$ up towards the neighbour above. In this way, on average, the magma is moving upward towards the volcano.

The dynamics at the volcano node are different: when $\:\:{Q}_{i}\left(t\right)<\:{Q}_{c}$, nothing happens, so the erupted volume is $\:\:e\left(t\right)=0$; when $\:\:{Q}_{i}\left(t\right)>\:{Q}_{c}$, only 1 unit of magma is erupted, so$\:\:e\left(t\right)=1$. The eruption and repose durations are computed as the time intervals during which $\:e\left(t\right)$ remain consecutively positive or null, respectively.

**Fig. 4:**
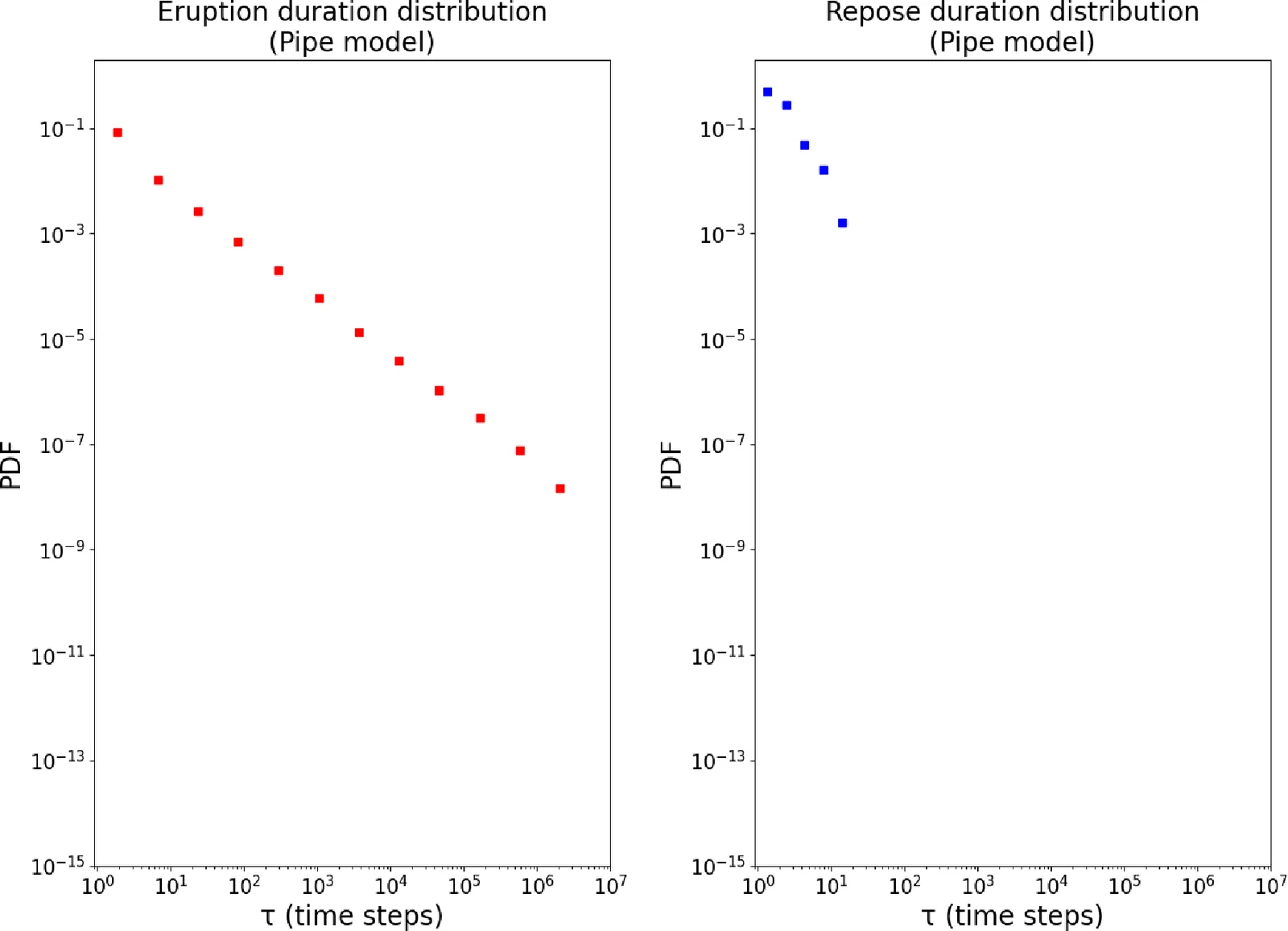
Eruption (left) and repose (right) duration distributions resulting from simulations of the pipe model with $\:{\mathrm{Q}}_{\mathrm{c}}=7,$ $\:\mathrm{n}=100$ and $\:\mathrm{T}=4\times\:1{0}^{6}$ time steps.

Regardless of the value of $\:\:{Q}_{c}$, the simulated distribution of eruption durations exhibits a power-law behaviour, while the produced repose periods are very short compared to the range of durations spanned by eruptions (Fig. S1). For instance, Fig. 4 shows the results of the pipe model run for $\:\:T=4\times\:{10}^{6}$ time steps, with $\:\:{Q}_{c}=7$ and * n=100*. The repose distribution is narrow, with a maximum duration of a few time steps, which means that the system is almost continuously erupting since the first eruptive episode. This is because, having a constant input $\:{I}_{0}=1\:$from below and an output $\:\:e\left(t\right)\:$that can be either * 0* or * 1* at each time step, the system tends to accumulate magma. Due to the threshold mechanism, this magma rapidly rises up and ends up being stored in the volcano node that will erupt for increasingly longer periods.

While the stochastic threshold model captures the behaviour of eruption durations, reproducing the variability of repose periods may require adding structural complexity to the system to allow magma to be stored and released at different paces.

In the following section, we introduce a rewiring method to obtain tree-like graphs from the pipe structure and show the results of running an adaptation of the pipe model to this new framework.

### From a pipe to trees

In order to deviate as little as possible from the original structure, we introduce a rewiring method, i.e., a recombination of the edges that keeps the number of nodes and edges constant, but changes the connectivity of the graph. This process is similar to the well-known Watts-Strogatz small-world model^26^, in which a rewiring method is applied to a regular ring lattice. In that model, each edge is randomly rewired with probability * p*. Tuning the parameter * p*, a regular graph (* p=0*) can be gradually turned into a random graph (* p=1*). For intermediate values of * p,* the graph exhibits small-world properties, namely the coexistence of high clustering, typical of regular graphs, and short average path lengths, typical of random graphs. Our method can transform the pipe structure into a tree-like graph, maintaining the connection between the source (* 0*) and the volcano (* n*). For each node $\:i\in\:\{1,\dots\:,n\}$ we rewire the edge (* i,i-1*) to a node * j<i* with a probability.

1 $$p_{i} (j) = \frac1{C_{i}} e^ {- \frac{i - j}D},\quad where \quad \,C_{i} =\sum_{{k < i}}e^ {- \frac{i - k}D}$$

**Fig. 5:**
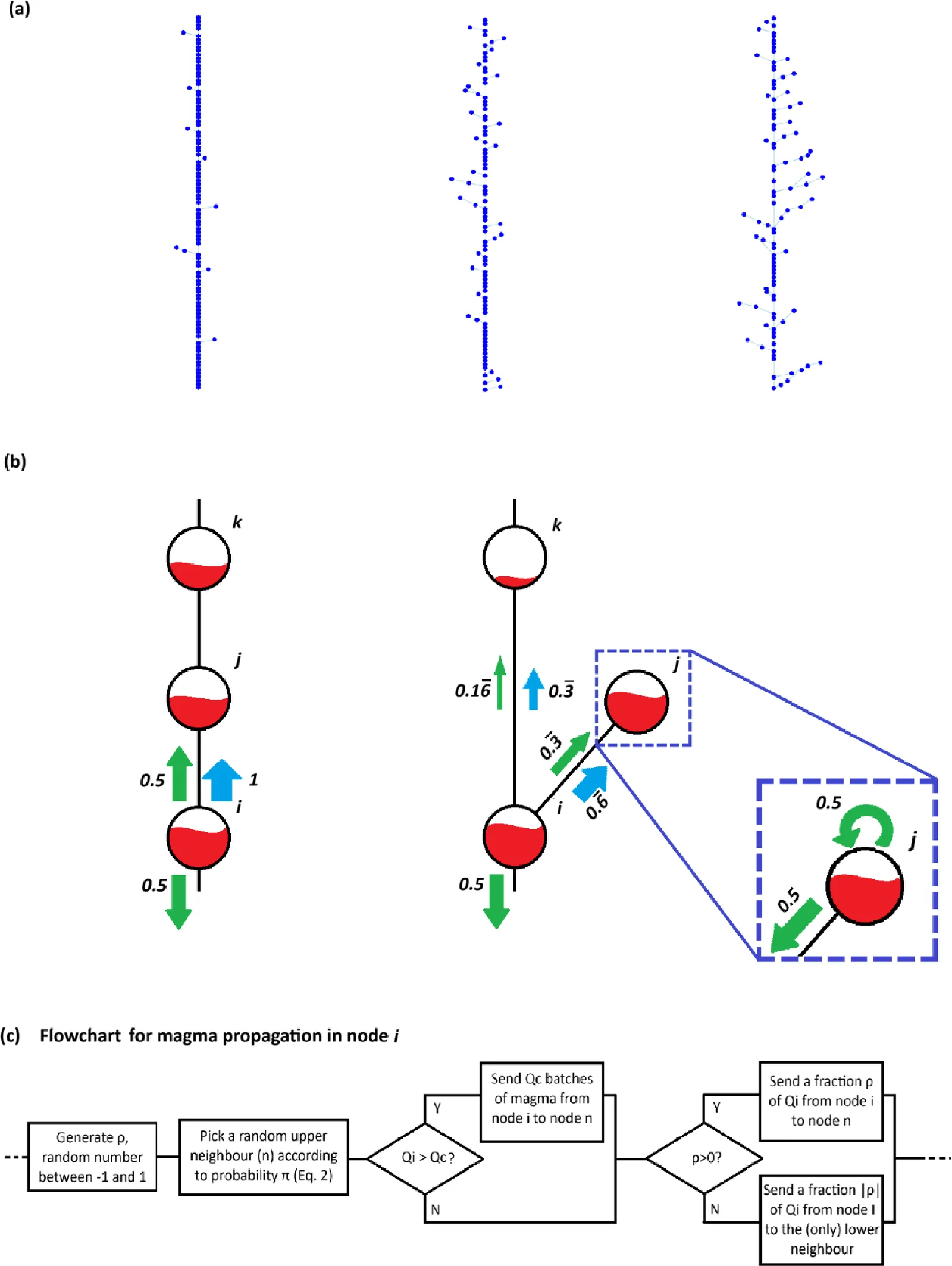
**(a)** Trees obtained through our rewiring method with branching parameters $\:\mathrm{D}=0.4$ (left), $\:\mathrm{D}=0.7$ (middle) and $\:\mathrm{D}=1.1$ (right). Notice the increasing presence and length of branches as $\:\mathrm{D}$ is increased. **(b)** Schematic representation of magma propagation from node i in presence of only one upper neighbour (left) and in presence of two upper neighbours (right) or no upper neighbours (blue box). The first case is what happens for every node in the pipe model, while in the tree model all three cases can occur. For instance, the situation on the right could arise from the situation on the left by rewiring the k-j edge to node i. The green arrows represent the possible movements of magma due to the stochastic part of the dynamics, while the blue arrows represent the possible movements of magma due to the threshold mechanism. The thickness of the arrows is proportional to the probability (reported in the scheme) of the movement they represent. **(c)** Flowchart for the algorithm that encodes the magma propagation mechanism.

* D* is a tunable parameter that controls the likelihood of connecting to nodes further back along the chain, allowing the creation of branches. The choice of the exponential decay in Eq. 1 of the probability with distance is the simplest way to allow for long-range connections while still promoting locality. It is not based on a specific physical law but acknowledges that close nodes are more likely to connect than distant ones in the same way that a close magma reservoir is more likely to trap a dyke than a distant one. In addition, it allows to introduce a characteristic scale (parameter $\:\:D$) that encodes the graphs’ structure and can be used to characterize the model outputs. The resulting network is a tree having a main path from the source to the volcano and sideways that deviate from the main path, which may represent storage regions (magma reservoirs) or alternative pathways that do not feed any eruption. Increasing the parameter $\:\:D$ increases the probability of creating longer and more intricate branches, as shown in Fig. 5a.

The pipe model must be slightly modified to run as a tree, in particular the stochastic process at nodes that don’t have an upper neighbour (leaves) or have more than one. In the tree model, in the absence of an upper neighbour, the magma does not move upwards, whereas in the presence of several upper neighbours, one of the upper neighbours is chosen through a weighted random choice. Given a node $\:\:i,$ we assign to each of its upper neighbours $\:j\in\:{N}_{up}\left(i\right)$ a probability.

  

2 $$\:{\pi\:}_{i}\left(j\right)=\frac{1}{{Z}_{i}}\frac{1}{j-i}\:,\quad where\quad {Z}_{i}={\sum\:}_{k\in\:{N}_{up}\left(i\right)}\frac{1}{k-i}$$

to receive the magma that should go upwards from node * i*.

This rule for magma redistribution, illustrated in the scheme of Fig. 5b and the flowchart of Fig. 5c, introduces a mechanism that penalizes its transport to more distant regions of the crust, that in nature may be due to dyke freezing^27^ and the physical presence of closer magma reservoirs that could also attract dykes by modifying the stress field around them^28^. The chosen functional form in Eq. 2, inversely proportional to the distance, may be interpreted as a simple representation of processes, such as buoyancy-driven dyke propagation^29^.

This addition assures the presence of multiple regions in the tree that tend to accumulate and release magma at different rates, depending on the local topology of the graph. This mechanism roughly mimics the formation and evolution of magma reservoirs in the crust and the role that they play in volcanic plumbing systems.

**Fig. 6:**
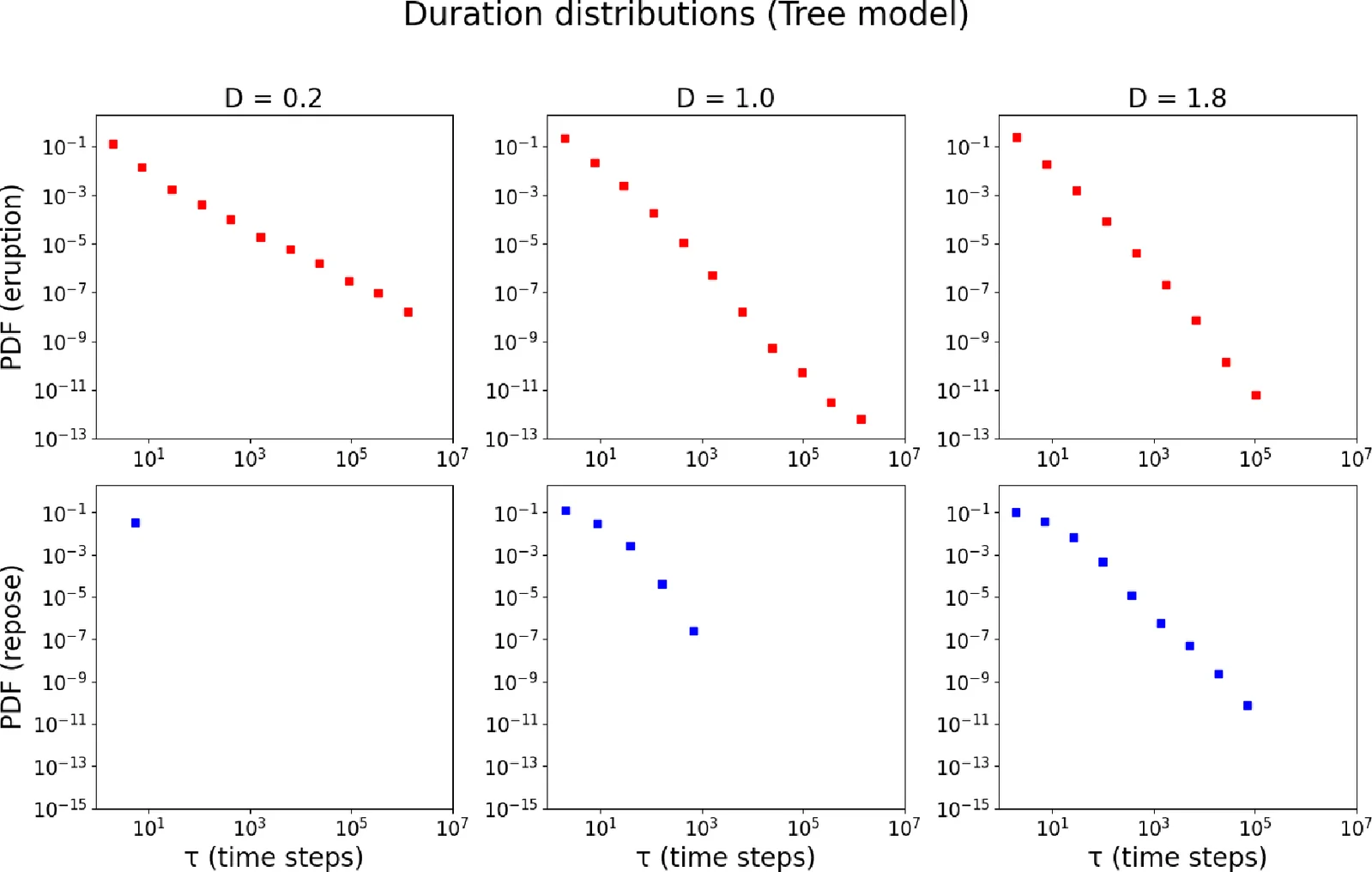
Simulated eruption (top) and repose (bottom) duration distributions resulting from the tree model, with $\:{\mathrm{Q}}_{\mathrm{c}}=7$ and three values of the branching parameter $\:\mathrm{D}$: from left to right, $\:\mathrm{D}$=$\:0.2,\:\mathrm{D}$=$\:1.0\:$and $\:\mathrm{D}$=* 1.8*. Notice the broadening of the repose duration distribution with the increasing of the parameter $\:\mathrm{D}$.

We ran simulations for different values of the branching parameter * D* and the threshold parameter $\:{Q}_{c}$, fixing the total simulation time to $\:T=4\times\:{10}^{6}$ time steps and the number of nodes * n=100* for computational feasibility. We also vary $\:{Q}_{c}$, which is a novelty with respect to the pipe model, to test whether in this new framework the model is sensitive to it. Figure 6 shows results for $\:{Q}_{c}=7$ and different values of the parameter $\:\:D$. With increasing * D*, the linear trend in log-log scale is retained for eruption durations, even though with a different slope, while the distribution of repose durations broadens and also acquires a scale-free tail.

**Fig. 7:**
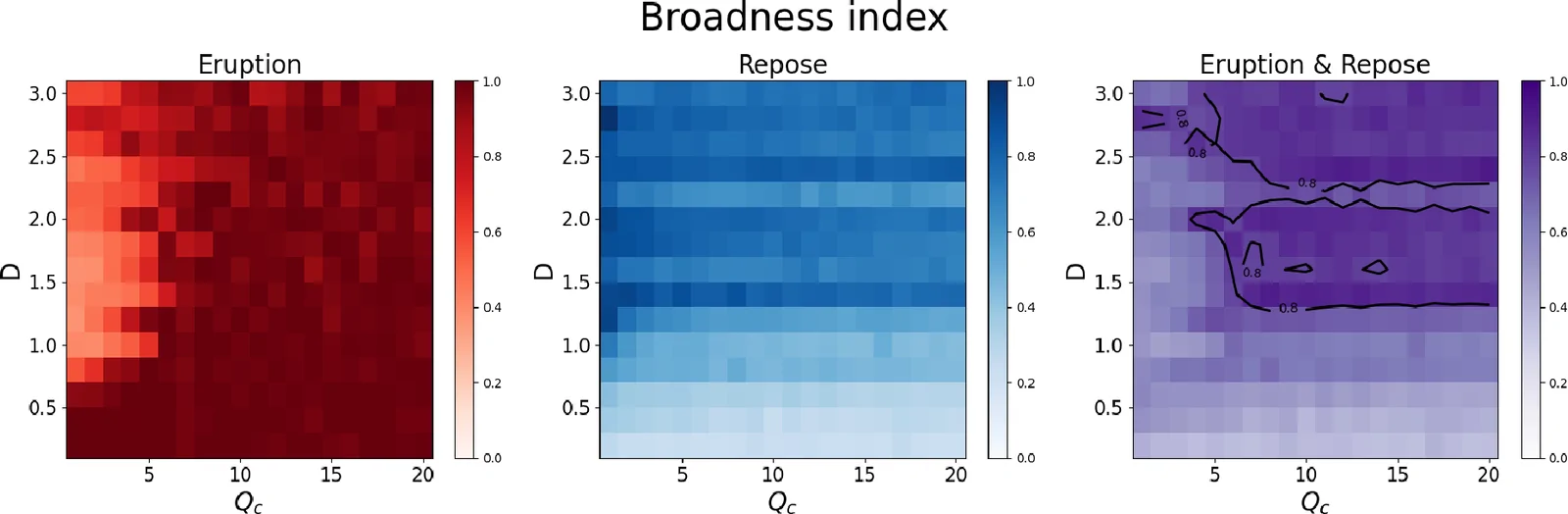
Broadness indices $\:\mathrm{B}$ for eruptions (left), repose periods (middle) and their harmonic mean (right) for different values of the branching parameter $\:\mathrm{D}$ and the threshold parameter $\:{\mathrm{Q}}_{\mathrm{c}}$. In the third plot, the high-B region described in the text (here highlighted by the $\:\mathrm{B}=0.8\:$line), in which both eruption and repose duration distributions are broad, is highlighted.

We were mainly interested to find the conditions for having broad distributions for both eruption durations and repose times. For this reason we introduced an index, the broadness index * B*, which represents how broad both simulated distributions are (see Methods section for its definition). Its value ranges between * 0* and * 1*, which would correspond to having very narrow or very broad distributions, respectively. Figure 7 shows the $\:\left({D,Q}_{c}\right)$-parameter space with the corresponding values of the broadness indices for eruptions, repose periods and their harmonic mean. If we stick to small values of the branching parameter * D*, the behaviour is similar to the pipe model: independently on the value of the threshold parameter, $\:{Q}_{c},\:$we obtain a broad distribution for eruption durations and only short repose times. However, if we stick to the original model with $\:{Q}_{c}=1,$ and increase the branching parameter, $\:D,\:$we lose the broadness of the eruption durations distributions while acquiring longer repose times. Similarly to what Watts & Strogatz did in their paper^26^, where they looked for a certain region of their parameter space where their rewired graph would exhibit the properties of a small-world network, we looked for a region of the parameter space in which we have high values of the joint broadness index, * B*. The region defined by $\:D\ge\:1.2$ and $\:{Q}_{c}\ge\:5$ is characterized by the highest values of * B*, ranging approximately between * 0.8* and * 0.9*. The values * D=1.2* and $\:{Q}_{c}=\:5$ also represent the zones of the parameter space characterized by the sharpest increase in * B* (Fig. S2), defining a transition of the model’s behaviour.

Finally, we fit the distributions in this high-* B* region with power-laws. The obtained exponents are $\:{\alpha\:}^{e}=2.28\:\pm\:\:0.16$ and $\:{\alpha\:}^{r}=2.67\:\pm\:\:0.29$, which are consistent with the values found from the statistical analyses of the dataset (Fig. 2a, c).

## Discussion

The analyses presented in this work show that both volcanic eruption and repose durations are characterized by broad, heavy-tailed distributions at medium-to-long timescales, in agreement with previous works^6,9^. A likelihood ratio test within the range of the power-law fit reveals no statistical preference between a log-normal and a power-law distribution, giving * p*-values * >0.1*. Looking over a wider range of durations ($\:\tau\:>300\:$ days), it turns out that the log-normal fit cannot be rejected (see Methods section) and is a better fit than the power-law (Fig. 3). As discussed above, the shape of the distributions is strongly constrained by the dataset. The longest durations are bounded by the finite observation window, being limited by the oldest event included in the analysis. Conversely, short durations are affected by uncertainties in defining the onset and termination of eruptions, as well as by the intrinsic limitation of the dataset, which excludes most repose periods shorter than three months. This rule, inherent in the dataset, has different effects on the two distributions. The effect on the repose times distribution is the least problematic, since it only introduces a lower cutoff which is already accounted for in the power-law fitting procedure through the computation of the minimal duration. A more complete dataset, including shorter repose intervals, could extend the analysis toward smaller values, without qualitatively altering the results presented here, since our statistical analysis focuses on the long-duration tail of the distribution. The major effect concerns the eruption durations distribution: two distinct successive eruptions of any duration could be merged into a single, longer eruption if they are separated by less than a 3-month period. This would lead to a possible overestimation of eruption durations, which is an important limitation of this dataset. Consequently, we do not conclude on the exact shape of the distribution between power-law and log-normal due to the incomplete nature of the dataset, this question being beyond the scope of this paper. Although the incompleteness of historical records and ambiguities in eruption definitions prevent a precise determination of the underlying statistical laws, the presence of heavy tails indicates a high variability in volcanic activity that cannot be adequately captured by simple Poissonian processes, so that long-lasting eruptions and extended repose periods, although rare, are significantly more frequent than would be expected under exponential distributions. This behaviour is consistent with complex volcanic systems in which magma transport, storage, and release occur over a wide range of interacting timescales. Within this framework, the network-based approach introduced here provides a simple yet effective way to explore how the structure of these volcanic systems can influence eruptive dynamics. By extending the original pipe model to tree-like graphs, we show that introducing branching pathways allows magma to be temporarily stored and released at different rates, naturally broadening the distribution of repose durations for sufficiently large values of the parameter * D*. The broadness of the eruption duration distributions is retained only in the case of sufficiently large values of the threshold parameter $\:{Q}_{c}$, which mimics an increased storage capacity or strength of the system before magma can be mobilized in larger quantities. This highlights a clear interplay between the structure of volcanic systems and threshold-driven processes that govern the magma flow. Our results suggest that the simultaneous emergence of broad eruption and repose duration distributions arises from the coupling of these two features. The system’s critical-like behaviour emerges naturally from local interactions under threshold rules within a branched topology, leading to heavy-tailed statistics across multiple timescales. The tree-like graphs used here demonstrate that modest structural complexity is sufficient to produce realistic distributions. However, more complex network architectures combined with processes that more closely mimic the physical behaviour of magma could offer further insights and potentially reproduce an even wider range of eruptive dynamics. Future work incorporating these features may further refine our understanding of the complexity of volcanic plumbing systems.

In conclusion, our results show that the topology of volcanic plumbing systems plays a fundamental role in shaping the statistical properties of eruptive behaviour. Therefore, representing these systems as networks could provide a powerful framework to capture their structural complexity and improve our understanding of how geometry and connectivity influence volcanic dynamics.

## Methods

### Data processing

We extrapolated eruption and repose durations from the Smithsonian Institution’s Global Volcanism Program (GVP) database^2^, updated to 2020. For the computation of the eruption durations, we considered only confirmed eruptions for which both the start and end years were reported. In the database, 33 eruptions were still ongoing at the time of data collection. We decided to keep them in the analysis in order not to lose statistical information on the tail of the distribution. For complete dates, we assigned an uncertainty of 1 day, unless an uncertainty was already provided in the dataset. When only the day was missing, we assigned the 15th of the month with an uncertainty of 15 days. When the month was missing, we assigned July 2nd with an error of 182 days. For the computation of repose durations, we included confirmed eruptions with missing end years, in order to account for all eruptive episodes in a volcano’s history and to avoid artificially long repose intervals. In these cases, the start year was used as a proxy for both the start and end of the eruption, which is reasonable since these events are usually associated with large temporal uncertainties that are accounted for in the data analysis. The uncertainty on the durations is obtained by summing up the uncertainties on the start and end dates.

To evaluate the impact of uncertainties associated with duration estimates, we performed a Monte Carlo analysis by generating multiple realizations of the dataset. For each realization, eruption and repose durations were independently sampled from a uniform distribution centered on the observed value, with bounds defined by the associated uncertainty.

For each simulated dataset, Cumulative Density Functions (CDFs) were computed using fixed logarithmic binning, ensuring consistency across realizations. The resulting ensemble of CDFs was then used to derive a mean distribution and its variability. The effect of uncertainties was quantified by calculating the pointwise relative deviation between the CDF obtained from the original dataset and the mean Monte Carlo CDF, and summarizing it through the mean relative deviation over all bins with non-zero probability density.

This analysis yields mean relative deviations of approximately 5%-10% for both distributions. These values indicate that the propagation of uncertainties introduces only limited modifications to the distributions. The observed differences remain small compared to the intrinsic variability of the data and do not alter the overall shape or scaling behaviour of the distributions.

In the plots of Figs. 1,2,4 and 6 we decided to show PDFs because they allow for a direct visual representation of the fitted power-law scaling exponent.

### Statistical analysis

To characterize the statistical properties of volcanic eruption and repose durations, we focused on heavy-tailed distributions, which are common in complex systems. In particular, we investigated whether these durations could be described by a power-law distribution, defined as

$$\:p\left(x\right)\propto\:\:{x}^{-\alpha\:},\:\:x>{x}_{min}$$

where $\:\alpha\:$ is the scaling exponent and $\:{x}_{min}$ is the lower bound above which the power-law behaviour holds. Power-law distributions are indicative of scale-free dynamics, meaning that events of different sizes follow the same statistical law without a characteristic scale.

We employed the Python package powerlaw^30^ to fit the observed distributions. The fitting procedure is based on maximum likelihood estimation (MLE), which provides robust estimates of the scaling exponent $\:\alpha\:$ for both discrete and continuous data. The method first determines the optimal $\:{x}_{min}$ that minimizes the distance between the empirical and model distributions, thereby focusing the fit on the heavy-tail region. The likelihood function is then maximized with respect to $\:\alpha\:$, yielding the most probable value given the observed data.

To assess the goodness of fit of the power-law model, we followed a Monte Carlo approach based on the method proposed by Clauset et al. (2009)^31^. After estimating the scaling exponent and the lower bound $\:{\tau\:}_{min}$, we computed the empirical Kolmogorov–Smirnov (KS) distance between the empirical data and the fitted power-law model. We then generated a large number of synthetic datasets, where values above $\:{\tau\:}_{min}$ were sampled from the fitted power-law distribution, while values below $\:{\tau\:}_{min}$ were randomly drawn from the empirical data. For each synthetic dataset, the fitting procedure was repeated and the corresponding synthetic KS distance was computed. The goodness-of-fit *p*-value was estimated as the fraction of synthetic KS values larger than the empirical one. A *p*-value greater than 0.1 is obtained for both eruption durations and repose times, which indicates that the power-law hypothesis cannot be rejected.

For power law fits, we performed Monte Carlo simulations in which only subsets of the dataset were retained in order to compute confidence intervals for the parameters $\:\alpha\:$ and $\:{\tau\:}_{min}$ (Fig. 2).

To compare the power-law model with alternative heavy-tailed distributions, we performed likelihood ratio tests using the implementation provided in the powerlaw^30^ package. In particular, we compared the power-law and log-normal (later defined) models by computing the log-likelihood ratio (R) and its associated* p*-value. In the case of a small *p*-value < 0.1, a positive value of R indicates that the data are better described by the power-law, whereas a negative value favours the log-normal model. In our case, the *p*-values for eruption durations and repose times are both > 0.1, so there is no preference between the two distributions.

Finally, we tested the goodness of fit of the log-normal distribution, which has probability density function

$$\:f\left(x\right)=\frac{1}{x\sigma\:\sqrt{2\pi\:}}\mathrm{exp}\left[-\frac{{\left(\mathrm{ln}x-\mu\:\:\right)}^{2}}{2{\sigma\:}^{2}}\right]$$

where $\:\mu\:$ and $\:\sigma\:$ are the scale and shape parameters, respectively, of the distribution. The agreement between the data and the log-normal model was assessed using the same method as for the power-law model. Again, *p*-values greater than 0.1 were found interpreted as an indication that the log-normal cannot be rejected.

The fitted parameters were $\:{\mu\:}^{e}=328$ and $\:{\sigma\:}^{e}=1.7$ for eruptions, with an average duration of * 1402* days ($\:\sim\:4$ years), and $\:{\mu\:}^{r}=992$ and $\:{\sigma\:}^{r}=1.9$ for repose times, with an average duration of * 6267* days ($\:\sim\:17$ years). The shape parameters, in particular, being relatively large, confirm the heavy-tailed nature of the distributions.

### The broadness index

To track how the shapes of the simulated distributions depend on the two parameters, we first compared the duration of the longest simulated event $\:{\tau\:}_{max}^{e,r}\left(D,{Q}_{c}\right)\:$ (* e* for eruption durations and * r* for repose times) to the total duration of the simulation * T*. We introduced the broadness index as the ratio between the logarithms of these quantities,

$$\:{B}^{e,r}(D,{Q}_{c})=\frac{\mathrm{l}\mathrm{o}\mathrm{g}\left({\tau\:}_{max}^{e,r}\right(D,{Q}_{c}\left)\right)}{\mathrm{l}\mathrm{o}\mathrm{g}\left(T\right)}\:$$

which is independent of the choice of the logarithmic base. Notice that $\:0\le\:B\le\:1$, since time is discrete and there cannot be durations longer than the simulation time, $\:1{\le\:\:\tau\:}_{max}^{e,r}\left(D,{Q}_{c}\right)\le\:T$. This normalization provides indices that don’t scale with the simulation time. Since we are interested in having both eruption and repose duration, for each pair $\:(D,{Q}_{c})$ we compute the harmonic mean between the eruption and repose broadness indices

$$\:B(D,{Q}_{c})=\frac{2}{\frac{1}{{B}^{e}(D,{Q}_{c})}\:+\:\frac{1}{{B}^{r}(D,{Q}_{c})}}$$

The harmonic mean provides a conservative measure of joint broadness, ensuring that high values are obtained only when both eruption and repose duration distributions are simultaneously broad.

## Supplementary Information

Below is the link to the electronic supplementary material.

Supplementary Material 1

## Data Availability

The .csv files with the eruption and repose durations used in the data analysis and the code written in Python to simulate the model are deposited on Zenodo. (https://doi.org/10.5281/zenodo.20067407)
