# Supplementary material for: A network-based approach to model volcanic repose durations
Source: Sci Rep. 2026 Jun 10;16:26307. doi: 10.1038/s41598-026-56253-7 (PMC13493914; doi:10.1038/s41598-026-56253-7)
Supplement: Supplementary file 1 [file 41598_2026_56253_MOESM1_ESM.docx]

**SUPPLEMENTARY FIGURES**

***Fig. S1:*** *Superposed eruption durations (left) and repose times (right) distributions resulting from simulations of the pipe model with values of* $Q_{c}$ *from* $1$ *to* $20$*,* $n=100$ *and* $T=4\times10^{6}$ *time steps. The transparency of the dots represents their density. Notice how the pipe model is not particularly sensitive to the threshold parameter: in all cases eruption durations exhibit a scale-free behaviour, while repose times are limited to small values.*

***Fig. S2:*** *Sensitivity of the joint broadness index,*$B,$*to the variation of the parameters, computed as the absolute value of the partial derivative of* $B$ *with respect to the threshold parameter, Qc, (left) and the branching parameter, D, (right), normalized by the respective maximum values. There is a sharp transition zone at approximately Qc=5 and D=1.2, followed by a plateau that corresponds to the high-B region identified in Fig. 7.*
